# Interventions on Adherence to Treatment in Children With Severe Asthma: A Systematic Review

**DOI:** 10.3389/fped.2018.00232

**Published:** 2018-08-21

**Authors:** Barbara Boutopoulou, Despoina Koumpagioti, Vasiliki Matziou, Kostas N. Priftis, Konstantinos Douros

**Affiliations:** ^1^Respiratory and Allergy Unit, 3rd Pediatric Department of National and Kapodistrian University of Athens, University General Hospital “Attikon”, Athens, Greece; ^2^2nd Pediatric Department of National and Kapodistrian University of Athens, “P & A Kiriakou” Children Hospital, Athens, Greece; ^3^Faculty of Nursing, National and Kapodistrian University of Athens, Athens, Greece

**Keywords:** children, severe asthma, difficult asthma, inhaled treatment, adherence

## Abstract

**Introduction:** Poor adherence to inhaled medication is a commonly encountered problem among children with asthma. However, there is a relatively paucity of data regarding the adherence of children with severe asthma, as well as the merit of any interventions to improve this adherence.

**Objectives:** The aim of this systematic review was to identify the available literature on the rate of adherence and the influence of interventions in improving adherence to controller inhaled medication, in children with severe asthma.

**Methods:** A systematic literature search was performed in MEDLINE/PubMed, Cochrane Library, and Scopus databases. Studies were included in the present review if their target population were children and/or adolescents with severe asthma and presented data on medication adherence before and after a given intervention.

**Results:** A total of seven studies, conducted in USA, Canada, and UK, and published between 2012 and 2018, met the inclusion criteria. Adherence to controller medication was assessed via either objective or subjective measures (questionnaires), or a combination of them. Interventions included communication during pediatric visits and audio-taped medical visits, individualized care programs, electronic monitoring devices, interactive website and peak–flow prediction with feedback. Adherence rates for the baseline (before intervention) or for the control groups ranged from 28 to 67%. In general, there was a significant improvement of adherence after intervention with rates increasing to 49–81%.

**Conclusion:** Adherence rate in children with severe asthma is not satisfactory but it can be improved after proper interventions. Nevertheless, the heterogeneity among adherence assessment tools, and the variety of interventions, in combination with the lack of studies focusing on severe asthma, highlight the need for further research in this field.

## Introduction

Asthma is a heterogeneous disease, usually characterized by chronic airway inflammation. It is defined by the history of respiratory symptoms such as wheeze, shortness of breath, chest tightness, and cough that vary over time and in intensity, together with variable expiratory airflow limitation (1). Asthma affects 1–18% of the population in different countries. It is one of the main causes of disability, health care services utilization, and quality of life impairment (2–5). It is estimated that about 14% of the children worldwide experience asthma symptoms. Asthma management aims to achieve good symptom control; maintain normal activities; minimize asthma attacks; reduce the side effects of treatment and as a result prevent the progression of obstructive lung damage during growth and then later in life (2).

Although the majority of asthma patients can be effectively controlled with the available medications, a substantial subset remains uncontrolled despite being offered the optimal therapy (6).

Severe asthma, according to European Respiratory Society (ERS)/American Thoracic Society (ATS) is asthma which requires Step 4 or 5 treatment [according to Global Initiative for Asthma (GINA) guidelines], e.g., high dose inhaled corticosteroids (ICS) and long-acting beta agonists (LABA) or leukotriene modifier/theophylline for the previous year or systemic ICS for ≥50% of the previous year to prevent it from becoming “uncontrolled” or which remains “uncontrolled” despite this therapy (6). Severe asthma includes patients with refractory or treatment-resistant asthma, or patients with incomplete response to treatment due to comorbidities (5). The primary approach of a child with problematic, severe respiratory symptoms, who is unresponsive to prescribed asthma therapy should be the confirmation of asthma diagnosis; secondly, one should explore if the child belongs to the category of “difficult-to-treat asthma” (2). The latter term is reserved for patients with ongoing factors that interfere with achieving good asthma control (allergen exposure, poor adherence), severe therapy-resistant asthma, asthma plus comorbidities (gastroesophageal reflux, obesity, obstructive apnea), or any combination of the above (7). Clinicians should also be aware of a prevalent cluster of chronic upper airway comorbidities, such as chronic rhinosinusitis and allergic rhinitis, which is recognized to patients with severe asthma but also seems that contribute to worsen asthma control and complicate asthma diagnosis and management (8). Distinguishing between severe asthma and uncontrolled asthma is crucial since the latter can be due to causes that can be more or less easily improved, such as the correction of a faulty inhaler technique and poor adherence (2, 9, 10).

Adherence is defined as the extent to which the patient's behavior matches the agreed recommendations from the prescriber. Patients can follow or not their doctors' recommendations, but failure to do so should not be a reason to blame the patient (11, 12). Adherence of asthmatics to long-term inhaled treatment has contributed substantial to asthma control and to morbidity reduction, yet, in general, it still remains suboptimal (13–16). The suboptimal adherence leads to poorer clinical outcomes and increased health care costs (17, 18).

Poor adherence (<60%) (19) to inhaled medication should be considered in all “difficult to control” patients. It has been reported that only 55% of children with moderate/severe persistent asthma use their controller medication daily (20).

Low adherence rates suggest an urgent need to increase adherence in order to reduce the burden of the disease. The improvement of adherence will result in better asthma control, and therefore, in a reduction of asthma severity (16, 21, 22). Shared decision making for medication/dose choice (23), inhaler reminders (24), home visits (25), prescribing ICS once daily versus twice (26), are all some of the interventions that are conducive to adherence improvement (2).

Although there are quite a few published reviews on the adherence of asthmatic children to controller medication and the effects of various interventions thereupon, there is still a lack of focus on severe asthma. Our aim in this systematic review was to identify the available literature on the rate of adherence and the influence of various interventions in improving adherence to controller inhaled medication, in children with severe asthma.

## Methods

This systematic review was based on the Preferred Reporting Items for Systematic Reviews and Meta—Analyses (PRISMA) statement, which aims to be the most accurate elaboration of a systematic review (27).

Two independent reviewers searched for publications in three of the most commonly used databases in medicine: Pubmed/Medline, Cochrane Library and Scopus. Key word combinations of “children,” “severe asthma,” “difficult asthma,” “inhaled treatment,” and “adherence,” were used to retrieve articles with these key words in title and abstract.

The criteria included were as follows:

1. Articles which were published from January of 2012 to March of 2018;
2. Articles written in English;
3. Studies that targeted children and/or adolescents;
4. Studies which have focused on severe asthma (as their main aim or as a subcategory of the study population); and
5. Studies on the effect of an intervention on adherence rate (as their main aim or as a subcategory of the study population).

### Data extraction

The data extraction was conducted by two reviewers. The characteristics collected for each study were references, sample characteristics, study design, duration, adherence assessment, intervention tools, and outcomes. Table 1 illustrates the main studies' characteristics.

**Table 1:** Study characteristics.

**References**	**Sample characteristics**	**Study design**	**Duration**	**Adherence assessment**	**Intervention tool**	**Outcomes**
Jochmann et al. (19)	93 children (STRA *n* = 21, Difficult asthma *n* = 51), 5–17 years, outpatient	Prospective observational cohort	6 months	Electronic monitoring device (smartinhaler) MARS-5 rating scale for self-reported adherence	Electronic monitoring	Median adherence for whole population was 74%.Good adherence (≥80%) in 42% of patients, Suboptimal adherence (< 80%) in 58% (*p* < 0.0065).
Feldman et al. (14)	192 children (severe persistent *n* = 52), 7–15 years (feedback group), 7–12 years (no feedback group), outpatient	Prospective longitudinal	6 weeks	Doser CT (MediTrack)	PEF prediction with feedback	Adherence to PEF feedback group 48.8 ± 4.5(%) and to no PEF feedback group 27.5 ± 4.9 (%) (*p* = 0.002).
Duncan et al. (29)	48 youth (severe persistent *n* = 8), 9–15 years, outpatient	Randomized controlled trial	5 months	Electronic monitoring device (MDILog-II)	Teamwork intervention (TI) Asthma education (AE) Standard care (SC)	Mean daily adherence for TI group (20-weeks) was 81%, while for the AE group 33.6% and for the SC group 37%.
Sleath et al. (28)	259 children (moderate/severe persistent *n* = 185), 8–16 years, outpatient	Prospective interventional	1 month	Questionnaire	Audio-taped medical visit and home visit interview	Children reported average control medication adherence was 72.4% (SD = 32.9; range, 0–100), while caregivers reported average control medication adherence 84.7% (SD = 26.1; range, 0–100).
Christakis et al. (31)	603 children (severe persistent *n* = 14), 2–10 years, outpatient	Randomized controlled trial	6 months	Questionnaire	Tailored interactive website	Controller medicine users with persistent asthma (intervention group) at both time points had significantly better adherence than the control group (*p* = 0.01).
Guénette et al. (32)	61 adolescents, 12–17 years (*N* = 349, aged 12–45 years)	Pragmatic controlled clinical trial	12 months	Morisky Medication Adherence Scale (MMAS −4) Medication Possession Rate (MPR)	Integrated care program	The integrated program had statistically significant effectiveness on ICS adherence, *p* = 0.0197 (mean MMAS–4 at baseline/12 month follow-up = 1.98/1.70, *p* = 0.051, mean MRP at baseline/12 month follow-up 19.41/22.86, *p* = 0.0629).
Ellis et al. (30)	167 adolescents, 12–16 years, outpatient	Randomized controlled trial	12 months	Medication Adherence subscale	Multisystemic Therapy- Health Care (MST-HC)	MST-HC was associated with better controller medication adherence at 6 months postintervention (*p* < 0.01).

*STRA, Severe therapy-resistant asthma; ICS, Inhaled Corticosteroids; PEF, Peak expiratory flow*.

## Results

### Studies selection

The search of the 3 databases retrieved 644 articles. Of these, 284 were duplicates and were excluded. The remaining 360 articles were screened for relevance. The full texts of 23 articles were assessed for eligibility; and finally 7 articles were chosen for the systematic review.

**Figure 1:**
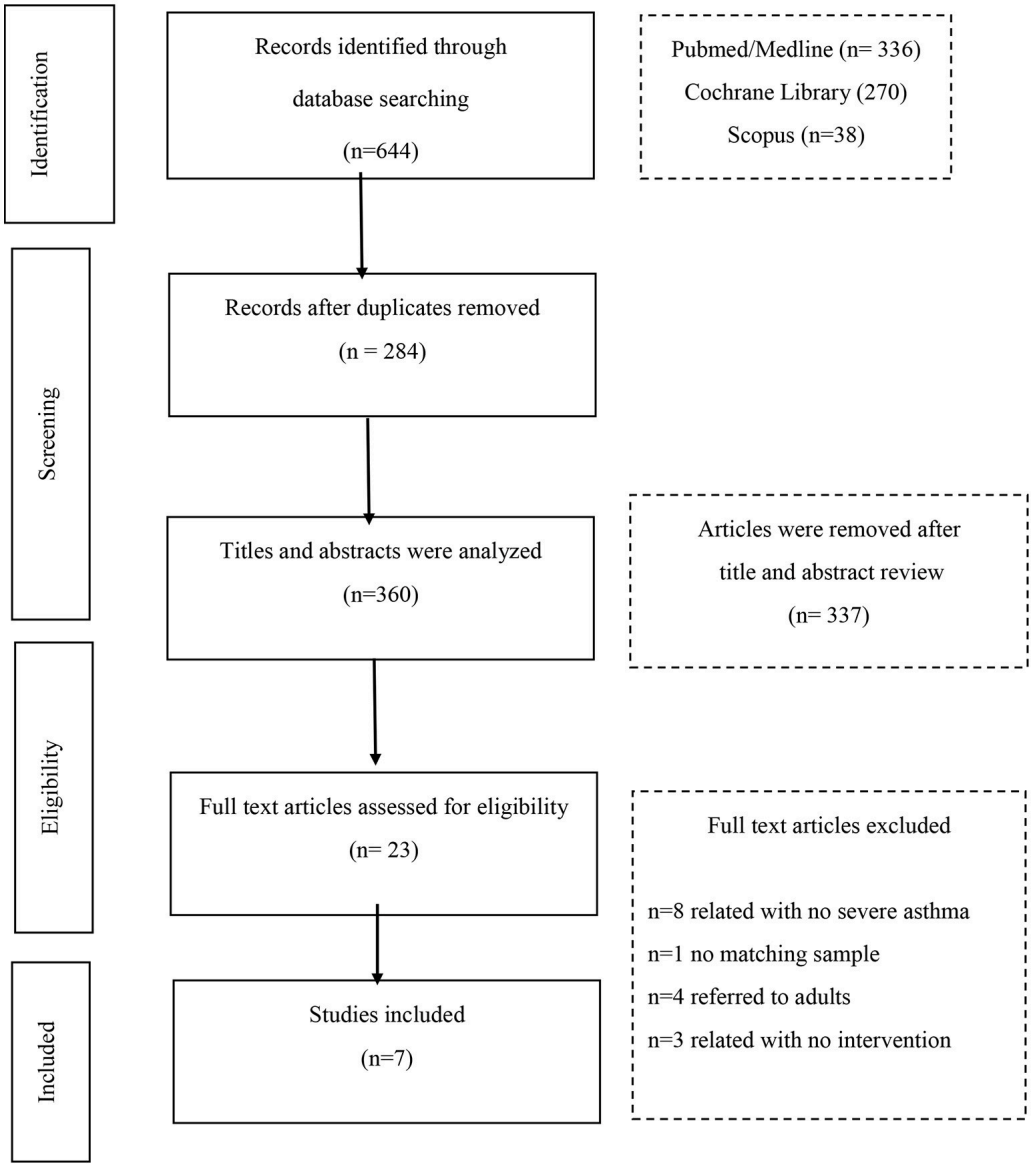
Flow diagram of studies included in systematic review.

The reasons for the exclusions are listed on the flow chart (Figure 1) which also provides the publication retrieval process.

### Studies description

Seven articles were included in the systematic review. Five of them were conducted in USA (14, 28–31); 1 in Canada (32); and 1 in UK (19).

Three studies were randomized controlled clinical trials (29–31), 1 was a controlled pragmatic clinical trial (32), 1 was a prospective observational cohort (19), 1 was a prospective longitudinal study (14), and 1 was a prospective interventional study (28).

Patients' recruitment was held mainly during outpatient visits. Guénette et al. (32) recruited patients with mediation of community pharmacists. Patients were children and their age ranged from 2 to 16 years old.

The total sample for severe asthma from the 7 studies was *n* = 508 children/adolescents, with the lowest sample size being *n* = 8 (29) and the highest *n* = 185 (28).

Severe asthma was a subcategory of the selected sample in all seven studies (14, 19, 28–32).

### Adherence assessment

Adherence in ICS treatment was evaluated through objective (14, 29), or subjective measures (28, 30, 31) or as a combination of both (19, 32). Objective measures of adherence assessment included electronic monitoring devices (Smartinhaler, Doser CT, MDILog-II) used in 4 of the studies (14, 19, 29, 32) and Medication Possession Ratio (MPR) used by Guénette et al. (32).

All three pre-mentioned devices developed as a canister attachment that fits on top of the majority of inhalers. They can provide accurate information on medication usage, including timing and number of doses taken, and all these recorded data can be used to guide asthma management (19).

Medication Possession Ratio (MPR) is a validated objective measure based on pharmacy records expressing the percentage of days supply received divided by a period of time and has been found to be more accurate than self-report (33).

Questionnaires are subjective measures for assessing adherence that are convenient and relatively unobtrusive and rely on self-report (34). The questionnaires used in the studies reported in this review were:

(1) Brief Medication Questionnaire, a tool for screening patients' adherence as well as their barriers to adherence. The tool includes a 5-item Regimen Screen that asks patients how they took each medication in the past week, a 2-item Belief Screen that asks about drug effects and bothersome features, and a 2-item Recall Screen about potential difficulties remembering (35);

(2) Morisky medication adherence scale (MMAS-4) which is a generic self-reported, medication-taking behavior scale using four questions about past medication use patterns (36);

(3) Medication Adherence Rating Scale (MARS-5) is a self-reported measure which evaluates both attitudes about medications and actual medication-taking behavior and consists of 10 items (37); and

(4) Medication Adherence subscale which measures adherence to controller medication (38).

### Intervention tools

Among the 7 studies, 3 had as a main research objective the increase of adherence rate after an intervention (28, 29, 31) whereas in the other 4 studies (14, 19, 30, 32) the adherence improvement rate was a subcategory of the findings.

Interventions design addressed solely the children (14, 19, 32), or there was also an element of parental involvement (28–31).

Briefly, Sleath et al. (28) audio-taped and coded communication during pediatric visits whether the provider included child or caregiver input into the asthma management treatment plan. Individualized care programs where health teams assess patients' and their caregivers' individual needs, share information and improve knowledge on asthma, were implemented in 3 of the studies (29, 30, 32). Christakis et al. (31) created a web-based tailored intervention aiming to increase children's positive beliefs about asthma management and Feldman et al. (14) used a piece of equipment where peak—flow prediction with feedback encouraged children to receive daily their inhaled treatment. Electronic monitoring devices were used in 1 study as an intervention (19) with the perception that adherence could improve following a period of monitoring.

The mean duration of interventions was 22 weeks, whereas the maximum (30, 32) and minimum duration (28) was 48 and 4 weeks, respectively.

### Outcomes

Across the 7 studies, adherence rates for the baseline (before intervention) or for the control groups ranged from 28 to 67% (14, 29, 31); there was a remarkable improvement after intervention with adherence rates increasing to 49–81% (14, 28, 29, 31).

Feldman et al. (14) found a significant positive difference between intervention and the control group (*p* = 0.02) using PEF prediction with feedback. Christakis et al. (31) using an interactive website, also showed a significant correlation between intervention and adherence improvement. Concerning the implementation of individualized care programs, Guénette et al. (32) showed statistically significant effectiveness measuring the adherence with MMAS-4 Scale (*p* = 0.0151). Statistically significant correlation was also demonstrated in Ellis et al. (30) measurements (*p* < 0.01). Team Intervention in Duncan et al. (29) study had a positive impact in adherence rate in comparison with Standard Care (81 vs. 37% respectively). Suboptimal adherence rates after intervention were presented in Jochman et al. (19) (median adherence was 74%) similarly with Sleath et al. (28) (average control medication adherence as reported by children was 72.4%).

## Discussion

This systematic review isolated seven articles from the recent literature, which focused on the improvement of adherence to inhaled medication after specific intervention, in children with severe asthma. Overall, the results of this systematic review highlight the importance of interventions in respect of adherence improvement, in children suffering from severe asthma.

Adherence can be assessed with objective or subjective measures (34). Objective measures used in some of the studies included in the current review were different kinds of electronic monitoring devices and the Medication Possession Ratio. There have also been included studies that used questionnaires for the subjective assessment of adherence. Some of the main objective adherence measuring tools for asthma medication adherence are the electronic monitoring devices, the canister weight, and the pharmacy refill data (39). Electronic monitoring has been labeled as the “gold standard” for assessing adherence due to the objective and detailed data it provides, but the cost and technology requirements (e.g., equipment, staff training) prohibit its widespread routine clinical use (40, 41). At the same time, some of the subjective measuring tools are interviews, questionnaires and diary/self-reporting (34). Subjective measures are inexpensive, convenient, and relatively unobtrusive and have the potential to provide information on related issues. Although, they mostly rely on self-report, their accuracy depends on psychometric properties and may mask variability of adherence across regimen components if assessed globally (40–42). Table 2 shows advantages and disavantages of methods for adherence assessment (34). As a great heterogeneity has appeared in asthma population regarding individualized capabilities, needs and preferences (43), researchers have concluded that more targeted and personalized methods of assessment are required (44).

**Table 2:** Summary of objective and subjective methods for adherence assessment (34).

**Objective methods**
**ELECTRONIC MONITORING**
**Advantages**
• Potentially measuring a variety of adherence behaviors (e.g., timing of dose, technique)
**Disadvantages**
• Usually not measuring actual consumption of medication
• Difficult to use
• Costly
• Associated with technological issues (e.g., battery failure and malfunction)
• Doubtful acceptability to patients and families
**PHARMACY REFILL DATA**
**Advantages**
• Inexpensive
• Fairly accurate (correlating with electronic monitoring data)
**Disadvantages**
• Not measuring consumption
• May be patients use other pharmacies, stockpiling medications, or family members' medications
• Difficulty in logistics (e.g., staff time, privacy regulations) to obtain records
**PILL COUNT/CANISTER WEIGHT**
A**dvantages**
• Inexpensive
• Fairly accurate (correlating with electronic monitoring data)
**Disadvantages**
• Patients may forget to bring their medication or miss their appointment
• May be manipulated by patient
• Not confirming that medication was taken
• Can be cumbersome for staff collecting and calculating
**Subjective methods**
**INTERVIEWS**
**Advantages**
• Can obtain extra information (e.g., regimen components, family issues), not only for medication use
• Can be administered over the telephone
**Disadvantages**
• Relies on self-report; subject to recall bias
• Psychometric properties and structure of the interview determine accuracy
**DIARY/SELF-MONITORING**
**Advantages**
• Reduces demands on memory
• Inexpensive
• Flexible-monitoring a range of variables in relation to a variety of adherence components
**Disadvantages**
• Relies on self-report; can be fabricated by patient
• Requires “adherence” to recording information, when adherence is a general concern
**QUESTIONNAIRES**
**Advantages**
• Inexpensive
• Convenient to be administered
**Disadvantages**
• Relies on self-report; subject to bias and social desirability
• May mask variability of adherence across regimen if assessed globally

Severe asthma in children is known to cause great morbidity, and raise asthma costs. The exact prevalence is unknown although it is estimated that 2–5% of asthmatic children have severe disease (45, 46). There is an interrelated relationship between severe asthma and adherence and clinicians can overestimate the severity of asthma if they do not assess adherence. Furthermore, poor adherence can lead to severe asthma if it is not corrected (47). According to our findings, adherence rate at the baseline for children with severe asthma ranged between 28 and 67%, which is in agreement with Celano et al. (48) who studied children with persistent asthma. The adherence of children with any kind of asthma is approximately 30–70% (49).

In our systematic review, we retrieved studies with interventions aimed at improving adherence. Some of the intervention tools that had been used were: home interviews and audio-taped medical visits (28); individualized care programs (29, 30, 32); electronic monitoring device use (19); interactive website (31) and peak–flow prediction with feedback. In another systematic review and meta-analysis various behavioral interventions, e.g., providing families with specific strategies to manage the regimen; educational interventions providing basic information to families about the patient's illness and the importance of adherence; organizational interventions such as introducing calendars for self-monitoring and facilitating discussion with caregivers about their child's illness or supporting caregiver-health care provider interactions were meta-analyzed and discussed (50). Furthermore, two other studies investigated the efficacy and safety of text messages for dose reminding (51, 52).

Asthma control is associated with adherence level. Jochmann et al. (19) found that children with poor adherence maintained poor control, while Ellis et al. (30) showed that children with asthma knowledge and controller device use skills had better medication adherence. Similarly, in other studies, adolescents report that it is more likely to adhere to treatment when they feel hopeful (53), view management tasks as important and feel competent (54), or when they intend to follow treatment recommendations (55, 56). Increased levels of self-efficacy are associated with better adherence, as well (57, 58). Adherence to ICS was an independent strong predictor of long term asthma control, with highest levels of asthma control found when the adherence raised above 80% of prescribed doses (16).

Our review showed that adherence rate in children with severe asthma can be increased after a proper intervention. In the studies included in our review, there was a significant increase of adherence rate, from 28–67% to 49–81%. There is a relatively lack of studies focusing on severe asthma, but the above findings are in agreement with the results of studies dealing with all kinds of asthma in children (24, 59). Although, the majority of studies have shown a positive effect, there are some instances where intervention with electronic asthma medication reminders did not improve the adherence rate (32, 51). Also, a scheduled follow up visit in combination with a comprehensive asthma management care program implemented in preschool children with asthma in the Netherlands did not correct adherence rates (60). A systematic review and meta-analysis found the studies that applied adherence education as intervention achieved a benefit of 20% points over control, while electronic trackers or reminders led to better adherence rates of 10% points. Researchers concluded that interventions' results depend on the group target, method and duration of intervention (61).

The main limitations of this review is the lack of studies focusing on severe asthma in children as most of the studies investigate adherence in children in the community and adult severe asthmatics rather than severely asthmatic children. Additionally, there is heterogeneity in the definitions of severe asthma among studies, a problem that has already been noticed by other researchers (62). Studies used different adherence assessment tools, therefore results were presented in various ways.

A weak point of all 7 studies is that it is not clear whether the adherence improvement was clinically meaningful. Literature suggests that in order to maintain asthma control, adherence rates have to be in excess of 80% (63).

## Conclusion

Adherence interventions have a positive impact on adherence rate in children with severe asthma. The remarkable heterogeneity between adherence assessment tools, and interventions, combined with the lack of studies focused on severe asthma, highlight the research gap in this field. There is a great need for further research focused exclusively on severe asthma and adherence treatment, in children.

## Author contributions

BB and DK contributed equally to this review. They did the literature research and the main writing of the article. VM, KP, and KD did all the academic support and the corrections during the whole process. KD did the finally fixing in English also.

### Conflict of interest statement

The authors declare that the research was conducted in the absence of any commercial or financial relationships that could be construed as a potential conflict of interest.
